# Supplementary material for: Analysis of Extracellular Vesicles in Gastric Juice from Gastric Cancer Patients
Source: Int J Mol Sci. 2019 Feb 22;20(4):953. doi: 10.3390/ijms20040953 (PMC6412909; doi:10.3390/ijms20040953)
Supplement: Supplementary file 1 [file ijms-20-00953-s001.pdf]

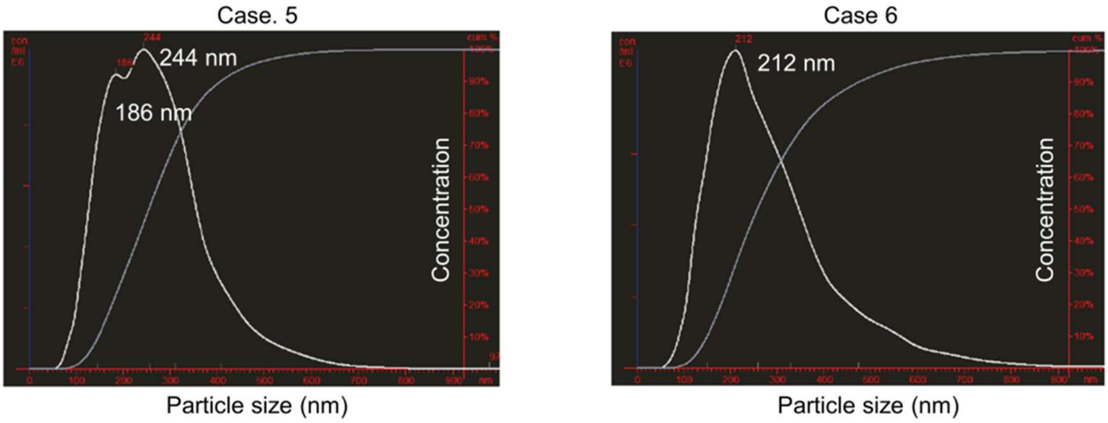

Figure S1. Size characterization of isolations from GJ by nanoparticle tracking analysis. The peak size of each sample is indicated in each graph. Four cases (cases 3–6) were examined. The graphs for cases 5 and 6 are shown.

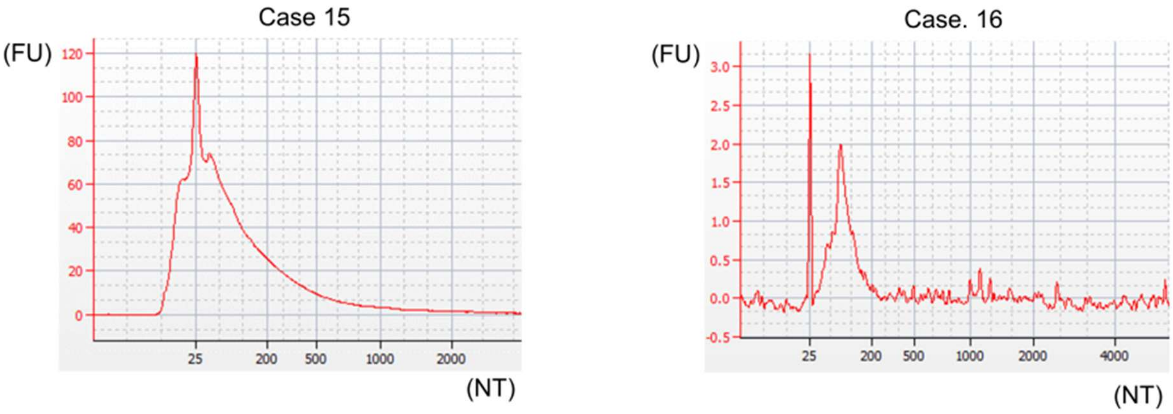

Figure S2. Characterization of RNA of EVs from GJ by use of the Bioanalyzer. Four cases (cases 13–16) of EVs from GJ were examined. Graphs of cases 15 and 16 are presented.

Control

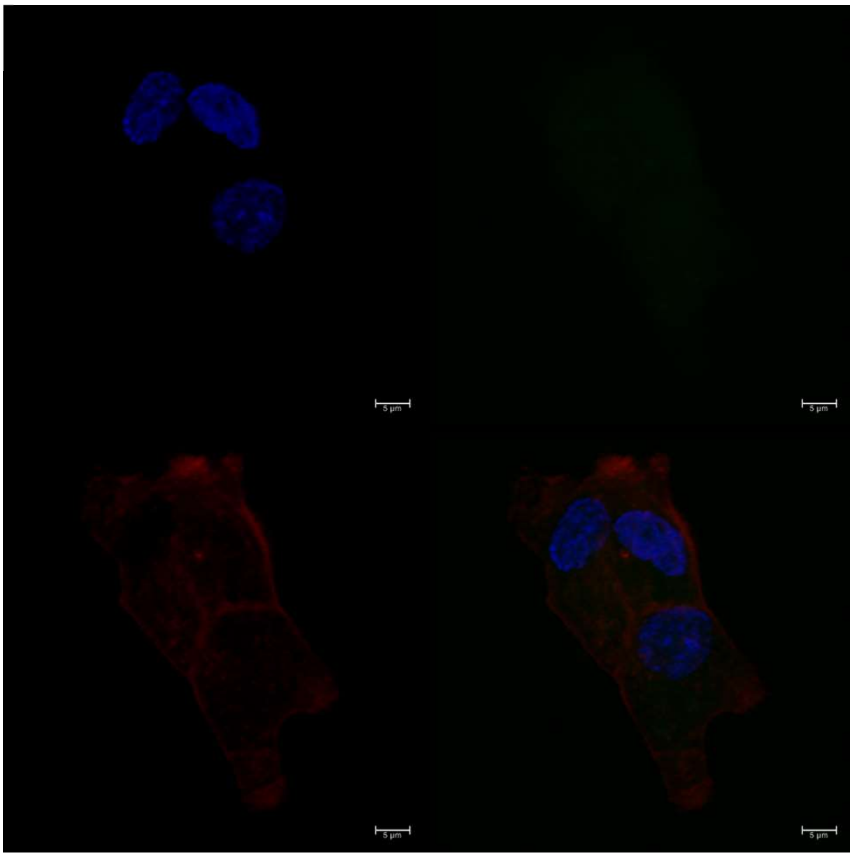

GJ-EVs

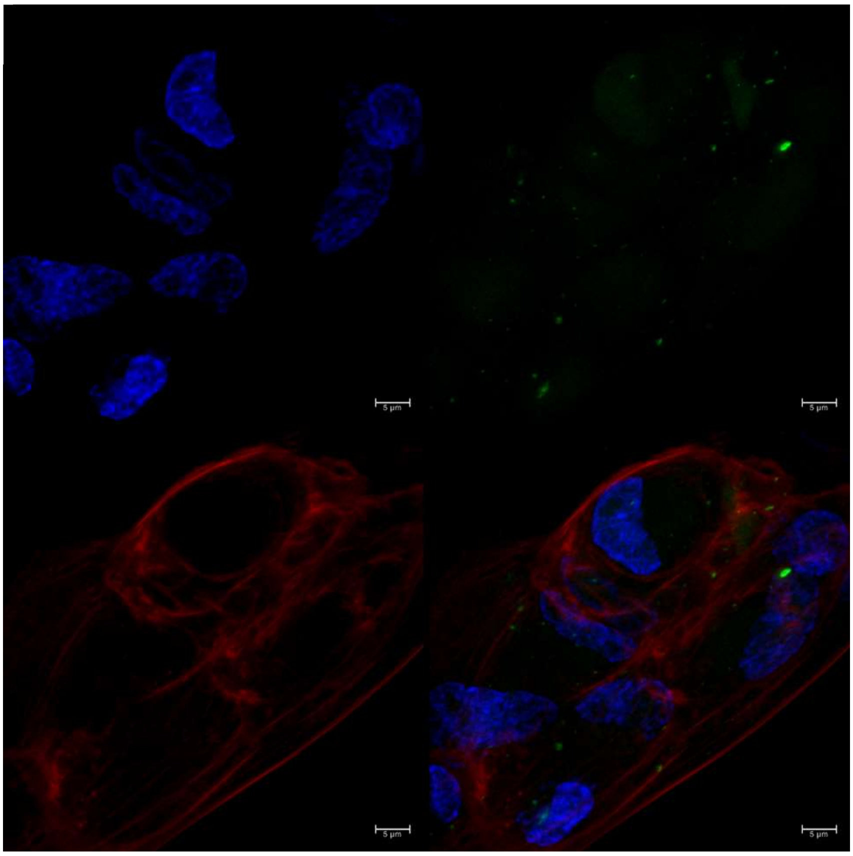

Figure S3. Immunofluorescence of GJ-EVs at 24 h after treatment of ASF-4 cells with GJ-EVs. **Upper** panels, treatment with control (PBS); **Lower** panels, treatment with GJ-EVs. RNA of GJ-EVs is dyed green; cell membrane, red; and nuclei, blue. Two cases of GJ-EVs were examined.
